# Supplementary material for: Competitive Match Running Speed Demands and Impact of Changing the Head Coach in Non-League Professional Football
Source: Sensors (Basel). 2025 Apr 30;25(9):2865. doi: 10.3390/s25092865 (PMC12539376; doi:10.3390/s25092865)
Supplement: Supplementary file 1 [file sensors-25-02865-s001.zip › sensors-3562948-supplementary.pdf]

**R script for packages, descriptive stats and LME**

Competitive Match Running Speed Demands and Impact of Changing the Head Coach in Non-League Professional Football

*Daniel T. Jackson et al. 2025*

```
#load packages
library(readxl)
library(ggplot2)
library(dplyr)
library(tidyr)
library(lme4)
library(lmtest)
library(clubSandwich)
library(sandwich)
library(lmtest)


#Load data e.g., dataset
data <- dataset
```

**Descriptive statistics**

```
# Descriptive statistics (mean and standard deviation) for all positions
 descriptive_overall <- data %>%
    summarise(
        Mean_TD= mean(TD, na.rm = TRUE),
        SD_TD= sd(TD, na.rm = TRUE),
        Mean_Walking = mean(walking, na.rm = TRUE),
        SD_Walking = sd(walking, na.rm = TRUE),
        Mean_Jogging = mean(jogging, na.rm = TRUE),
        SD_Jogging = sd(jogging, na.rm = TRUE),
        Mean_Running = mean(Running, na.rm = TRUE),
        SD_Running = sd(Running, na.rm = TRUE),
        Mean_HSRD = mean(HSRD, na.rm = TRUE),
        SD_HSR = sd(HSRD, na.rm = TRUE),
        Mean_Sprint = mean(sprint, na.rm = TRUE),
        SD_Sprint = sd(sprint, na.rm = TRUE)
    )

 print(descriptive_overall)

# Descriptive statistics (mean and standard deviation) for each position
descriptive_by_position <- data %>%
  group_by(position) %>%
  summarise(
Mean_TD= mean(TD, na.rm = TRUE),
        SD_TD= sd(TD, na.rm = TRUE),
        Mean_Walking = mean(walking, na.rm = TRUE),
        SD_Walking = sd(walking, na.rm = TRUE),
        Mean_Jogging = mean(jogging, na.rm = TRUE),
        SD_Jogging = sd(jogging, na.rm = TRUE),
```

```
      Mean_Running = mean(Running, na.rm = TRUE),
      SD_Running = sd(Running, na.rm = TRUE),
      Mean_HSRD = mean(HSRD, na.rm = TRUE),
      SD_HSR = sd(HSRD, na.rm = TRUE),
      Mean_Sprint = mean(sprint, na.rm = TRUE),
      SD_Sprint = sd(sprint, na.rm = TRUE)
  )

print(descriptive_by_position)
```

**Simple linear models**

```
# simple linear model with position, compute AIC, BIC, and RMSE
compute_position_model_metrics <- function(response_variable, data, model_name) {
# Fit the model
lm_model <- lm(as.formula(paste(response_variable, "~ position")), data = data)
 # Compute AIC, BIC, and RMSE
  aic_value <- AIC(lm_model)
  bic_value <- BIC(lm_model)
  residuals <- resid(lm_model)
  rmse_value <- sqrt(mean(residuals^2))
   #print results
  cat("\nLinear Model with Position -", model_name, "\n")
  cat("AIC:", aic_value, "\n")
  cat("BIC:", bic_value, "\n")
  cat("RMSE:", rmse_value, "\n")
  cat("---------------------------------------\n")
  return(lm_model)
}

# Apply function for each running metric
lm_position_TD <- compute_position_model_metrics("TD", data, "Total Distance")
lm_position_walking <- compute_position_model_metrics("walking", data, "Walking")
lm_position_jogging <- compute_position_model_metrics("jogging", data, "Jogging")
lm_position_running <- compute_position_model_metrics("Running", data, "Running")
lm_position_HSRD <- compute_position_model_metrics("HSRD", data, "High-Speed Running
Distance")
lm_position_sprinting <- compute_position_model_metrics("sprint", data, "Sprinting")
```

For each lm with single variable, there was poorer model fit indicies for each running metric when compared to multivariate models.

**Fit linear models**

```
#convert match outcome to factor "win" as reference:
data$outcome <- factor(data$outcome, levels = c("W", "D", "L"))

# Fit linear models for each running speed category with coach, position, and outcome as the
predictor
lm_model_TD <- lm(TD ~ coach + position + outcome, data = data)
```

```
lm_walking <- lm(walking ~ coach + position + outcome, data = data)
lm_jogging <- lm(jogging ~ coach + position + outcome, data = data)
lm_running <- lm(Running ~ coach + position + outcome, data = data)
lm_HSRD <- lm(HSRD ~ coach + position + outcome, data = data)
lm_sprint <- lm(sprint ~ coach + position + outcome, data = data)


# Diagnostic plots
# Check residuals for linear model (optional diagnostic)
par(mfrow = c(2, 2))
plot(lm_model_TD)
#complete for each running speed model as above
```

#several lm show heteroscedasticity/non-normality at tails. Consideration of robust standard errors or to transform variables. To allow for better interpretation and keep results consistent robust SE were chosen.

As was observed in simple lm, models with less demonstrated variables poorer fit.

**MIXED EFFECTS MODELS**

```
# fit mixed effects model for TD
mixed_model_TD <- lmer(TD ~ coach + position + outcome + (1 | `game`), data = data)
summary(mixed_model_TD)

# fit mixed effects model for walking
mixed_walking <- lmer(walking ~ coach + position + outcome + (1 | `game`), data = data)
summary(mixed_walking)

# fit mixed effects model for jogging
mixed_jogging <- lmer(jogging ~ coach + position + outcome + (1 | `game`), data = data)
summary(mixed_jogging)

#fit mixed effects model for running
mixed_running <- lmer(Running ~ coach + position + outcome + (1 | `game`), data = data)
summary(mixed_running)

#mixed effects model for HSR
mixed_HSRD <- lmer(HSRD ~ coach + position + outcome + (1 | `game`), data = data)
summary(mixed_HSRD)

# mixed effects model for sprint
mixed_sprint <- lmer(sprint ~ coach + position + outcome + (1 | `game`), data = data)
summary(mixed_sprint)



#Model assumptions


# residuals vs fitted linearity and homoscedasticity
```

```
plot(mixed_model_TD)
plot(mixed_walking)
plot(mixed_jogging)
plot(mixed_running)
plot(mixed_HSRD)
plot(mixed_sprint)

#Q-Q plot normality of residuals
# Extract residuals from the mixed model
residuals_mixedTD <- resid(mixed_model_TD)
qqnorm(residuals_mixedTD)
qqline(residuals_mixedTD, col = "red")

residuals_mixedwalking <- resid(mixed_walking)
qqnorm(residuals_mixedwalking)
qqline(residuals_mixedwalking, col = "red")

residuals_mixedjogging <- resid(mixed_jogging)
qqnorm(residuals_mixedjogging)
qqline(residuals_mixedjogging, col = "red")

residuals_mixedrunning <- resid(mixed_running)
qqnorm(residuals_mixedrunning)
qqline(residuals_mixedrunning, col = "red")

residuals_mixedHSRD <- resid(mixed_HSRD)
qqnorm(residuals_mixedHSRD)
qqline(residuals_mixedHSRD, col = "red")

residuals_mixedsprint <- residuals(mixed_sprint)
qqnorm(residuals_mixedsprint)
qqline(residuals_mixedsprint, col = "red")


# Scale location plot homoscedasticity
# Extract fitted values
fitted_values <- fitted(mixed_model_TD) # Replace with your model object
plot(fitted_values, sqrt(abs(residuals_mixed)),
    main = "Scale-Location Plot",
    xlab = "Fitted Values",
    ylab = "Square Root of |Residuals|")
abline(h = 0, col = "red")

#demonstrates some mild heteroscedasticity and non-normality
#use robust standard errors across both lm and mixed effect models


#model fit

# Calculate AIC
aic_mixed_TD <- AIC(mixed_model_TD)
```

```
cat("AIC (mixed_model_TD):", aic_mixed_TD, "\n")

# Calculate BIC
bic_mixed_TD <- BIC(mixed_model_TD)
cat("BIC (mixed_model_TD):", bic_mixed_TD, "\n")

# Calculate RMSE
# Extract residuals
residuals_mixed_TD <- resid(mixed_model_TD)
rmse_mixed_TD <- sqrt(mean(residuals_mixed_TD^2))
cat("RMSE (mixed_model_TD):", rmse_mixed_TD, "\n")



#function to compare AIC, BIC, and RMSE for linear and mixed models:
compare_aic_bic_rmse <- function(lm_model, mixed_model, model_name) {
  # lm metrics
  aic_lm <- AIC(lm_model)
  bic_lm <- BIC(lm_model)
  rmse_lm <- sqrt(mean(resid(lm_model)^2))

  #mixed model metrics
  aic_mixed <- AIC(mixed_model)
  bic_mixed <- BIC(mixed_model)
  rmse_mixed <- sqrt(mean(resid(mixed_model)^2))

  #print results
  cat("\nModel Comparison for:", model_name, "\n")
  cat("Linear Model - AIC:", aic_lm, "BIC:", bic_lm, "RMSE:", rmse_lm, "\n")
  cat("Mixed Model - AIC:", aic_mixed, "BIC:", bic_mixed, "RMSE:", rmse_mixed, "\n")
  cat("--------------------------------------------------\n")
}

# apply to models:
compare_aic_bic_rmse(lm_model_TD, mixed_model_TD, "Total Distance")
compare_aic_bic_rmse(lm_walking, mixed_walking, "Walking")
compare_aic_bic_rmse(lm_jogging, mixed_jogging, "Jogging")
compare_aic_bic_rmse(lm_running, mixed_running, "Running")
compare_aic_bic_rmse(lm_HSRD, mixed_HSRD, "High-Speed Running Distance")
compare_aic_bic_rmse(lm_sprint, mixed_sprint, "Sprinting")
```

```
# Function to compare robust SE results for linear and mixed models:
compare_robust_results <- function(lm_model, mixed_model, model_name) {
  cat("\n", "Model Comparison with Robust SE for:", model_name, "\n")
  cat("==============================\n")

  # Robust SE for Linear Model
  cat("\nLinear Model with Robust SE:\n")
  robust_se_lm <- coeftest(lm_model, vcov = vcovHC(lm_model, type = "HC1"))
  print(robust_se_lm)

  # Robust SE for Mixed Model
  cat("\nMixed Effects Model with Robust SE:\n")
  robust_se_mixed <- coef_test(mixed_model, vcov = "CR2")
  print(robust_se_mixed)

  cat("\n--------------------------------------------------\n")
}

# Apply the function to all running metrics
compare_robust_results(lm_model_TD, mixed_model_TD, "Total Distance")
compare_robust_results(lm_walking, mixed_walking, "Walking")
compare_robust_results(lm_jogging, mixed_jogging, "Jogging")
compare_robust_results(lm_running, mixed_running, "Running")
compare_robust_results(lm_HSRD, mixed_HSRD, "High-Speed Running Distance")
compare_robust_results(lm_sprint, mixed_sprint, "Sprint")
```

RESULTS FROM R OUTPUT

Summary for Total Distance :
Linear Model - AIC: 4940.37 , BIC: 4974.028 , RMSE: 661.7181
Mixed Model - AIC: 4838.531 , BIC: 4875.929 , RMSE: 585.7479
Summary for Walking :
Linear Model - AIC: 4451.518 , BIC: 4485.176 , RMSE: 301.5402
Mixed Model - AIC: 4374.437 , BIC: 4411.835 , RMSE: 282.4487
Summary for Jogging :
Linear Model - AIC: 4797.058 , BIC: 4830.716 , RMSE: 525.5443
Mixed Model - AIC: 4713.367 , BIC: 4750.765 , RMSE: 499.9763
Summary for Running :
Linear Model - AIC: 4445.535 , BIC: 4479.193 , RMSE: 298.6538
Mixed Model - AIC: 4372.236 , BIC: 4409.634 , RMSE: 287.0674
Summary for High-Speed Running Distance :
Linear Model - AIC: 3859.16 , BIC: 3892.818 , RMSE: 116.345
Mixed Model - AIC: 3800.037 , BIC: 3837.435 , RMSE: 111.1003
Summary for Sprinting :
Linear Model - AIC: 3409.693 , BIC: 3443.351 , RMSE: 56.48327
Mixed Model - AIC: 3364.096 , BIC: 3401.494 , RMSE: 55.16732

Model Comparison with Robust SE for: Total Distance
========================================================

Linear Model with Robust SE:

t test of coefficients:

```
             Estimate Std. Error  t value  Pr(>|t|)
(Intercept) 10390.025     79.201 131.1862 < 2.2e-16 ***
coachb       -358.860    133.590  -2.6863  0.007624 **
coachc       -512.965     87.056  -5.8924 1.013e-08 ***
positionMF    415.286     92.175   4.5054 9.469e-06 ***
positionST   -166.307    133.567  -1.2451  0.214051
positionWD    567.921     98.288   5.7781 1.874e-08 ***
outcomeD       86.098    109.884   0.7835  0.433927
outcomeL       31.599     82.978   0.3808  0.703609
---
Signif. codes:  0 '***' 0.001 '**' 0.01 '*' 0.05 '.' 0.1 ' ' 1
```

Mixed Effects Model with Robust SE:

```
       Coef. Estimate    SE  t-stat d.f. (Satt) p-val (Satt) Sig.
 (Intercept)  10393.9 103.6 100.336       17.96       <0.001  ***
      coachb   -364.6 183.8  -1.984        4.44        0.111
      coachc   -505.3 107.9  -4.683       18.25       <0.001  ***
  positionMF    410.3  82.4   4.978       39.09       <0.001  ***
  positionST   -155.4 121.7  -1.276       32.10        0.211
  positionWD    560.4  80.7   6.942       38.25       <0.001  ***
```

```
   outcomeD    86.5 167.8   0.516       14.18        0.614
   outcomeL    25.2 126.8   0.198       25.66        0.844

--------------------------------------------------------
> compare_robust_results(lm_walking, mixed_walking, "Walking")

 Model Comparison with Robust SE for: Walking
========================================================

Linear Model with Robust SE:

t test of coefficients:

            Estimate Std. Error t value  Pr(>|t|)
(Intercept) 3984.5166    40.8030 97.6526 < 2.2e-16 ***
coachb        60.5846    57.6491  1.0509   0.29413
coachc        67.3370    39.5353  1.7032   0.08955 .
positionMF   -75.3413    40.1634 -1.8759   0.06163 .
positionST   143.3816    74.8671  1.9151   0.05641 .
positionWD  -184.7651    42.6828 -4.3288  2.04e-05 ***
outcomeD      58.5841    53.0051  1.1053   0.26993
outcomeL      -8.1879    36.8941 -0.2219   0.82452
---
Signif. codes:  0 ‘***’ 0.001 ‘**’ 0.01 ‘*’ 0.05 ‘.’ 0.1 ‘ ’ 1


Mixed Effects Model with Robust SE:
      Coef. Estimate   SE t-stat d.f. (Satt) p-val (Satt) Sig.
 (Intercept)  3986.14 43.1 92.538       19.47       <0.001  ***
     coachb    59.07 64.4  0.918        4.42       0.4061
     coachc    67.77 52.8  1.283       18.23       0.2155
  positionMF   -77.84 37.5 -2.078       39.12       0.0443    *
  positionST   141.24 65.9  2.144       32.26       0.0396    *
  positionWD  -184.32 33.0 -5.590       38.27       <0.001  ***
   outcomeD    54.39 66.4  0.819       14.25       0.4260
   outcomeL    -8.88 48.6 -0.183       25.64       0.8565

--------------------------------------------------------
> compare_robust_results(lm_jogging, mixed_jogging, "Jogging")

 Model Comparison with Robust SE for: Jogging
========================================================

Linear Model with Robust SE:

t test of coefficients:

           Estimate Std. Error t value  Pr(>|t|)
(Intercept) 4464.613     66.168 67.4738 < 2.2e-16 ***
coachb      -193.731     80.371 -2.4104  0.016528 *
coachc      -203.319     71.756 -2.8335  0.004914 **
```

```
positionMF   154.098     70.181  2.1957  0.028869 *
positionST  -445.357    108.243 -4.1144 5.007e-05 ***
positionWD   362.302     83.311  4.3488 1.872e-05 ***
outcomeD    -134.936     82.964 -1.6264  0.104894
outcomeL     -94.962     68.313 -1.3901  0.165520
---
Signif. codes:  0 ‘***’ 0.001 ‘**’ 0.01 ‘*’ 0.05 ‘.’ 0.1 ‘ ’ 1


Mixed Effects Model with Robust SE:
      Coef. Estimate    SE t-stat d.f. (Satt) p-val (Satt) Sig.
 (Intercept)   4467.0  68.0  65.73       20.00       <0.001  ***
     coachb   -194.1 102.9  -1.89        4.41       0.1258
     coachc   -200.0  84.1  -2.38       18.22       0.0285    *
  positionMF    153.8  63.0   2.44       39.13       0.0193    *
  positionST   -442.7  95.3  -4.64       32.32       <0.001  ***
  positionWD    357.8  74.7   4.79       38.28       <0.001  ***
   outcomeD   -134.2  89.1  -1.51       14.28       0.1540
   outcomeL    -97.5  84.1  -1.16       25.63       0.2569

--------------------------------------------------------
> compare_robust_results(lm_running, mixed_running, "Running")

 Model Comparison with Robust SE for: Running
============================================================

Linear Model with Robust SE:

t test of coefficients:

           Estimate Std. Error t value  Pr(>|t|)
(Intercept) 1552.561     44.542 34.8558 < 2.2e-16 ***
coachb      -167.724     60.501 -2.7723 0.0059116 **
coachc      -297.856     35.129 -8.4789 1.012e-15 ***
positionMF   159.112     44.089  3.6089 0.0003596 ***
positionST  -110.440     52.824 -2.0907 0.0373865 *
positionWD   144.681     46.961  3.0809 0.0022531 **
outcomeD     110.684     48.787  2.2687 0.0239880 *
outcomeL      84.977     38.412  2.2122 0.0276957 *
---
Signif. codes:  0 ‘***’ 0.001 ‘**’ 0.01 ‘*’ 0.05 ‘.’ 0.1 ‘ ’ 1


Mixed Effects Model with Robust SE:
      Coef. Estimate   SE t-stat d.f. (Satt) p-val (Satt) Sig.
 (Intercept)   1551.6 48.4  32.03        20.4       <0.001  ***
     coachb   -169.2 76.8  -2.20         4.4       0.0863    .
     coachc   -297.8 38.4  -7.75        18.2       <0.001  ***
  positionMF    161.0 44.4   3.63        39.1       <0.001  ***
  positionST   -106.2 43.7  -2.43        32.4       0.0209    *
  positionWD    146.0 40.8   3.58        38.3       <0.001  ***
```

```
   outcomeD    111.8 54.0   2.07        14.3       0.0570    .
   outcomeL     84.2 46.1   1.83        25.6       0.0795    .

--------------------------------------------------------
> compare_robust_results(lm_HSRD, mixed_HSRD, "High-Speed Running Distance")

 Model Comparison with Robust SE for: High-Speed Running Distance
============================================================

Linear Model with Robust SE:

t test of coefficients:

            Estimate Std. Error t value  Pr(>|t|)
(Intercept)  327.636     13.217 24.7889 < 2.2e-16 ***
coachb       -42.131     25.138 -1.6760 0.0947724 .
coachc       -58.004     15.202 -3.8155 0.0001647 ***
positionMF   123.857     16.555  7.4817 8.001e-13 ***
positionST   142.653     22.034  6.4741 3.838e-10 ***
positionWD   169.534     17.037  9.9509 < 2.2e-16 ***
outcomeD      59.536     19.525  3.0492 0.0024971 **
outcomeL      42.939     14.503  2.9607 0.0033116 **
---
Signif. codes:  0 '***' 0.001 '**' 0.01 '*' 0.05 '.' 0.1 ' ' 1


Mixed Effects Model with Robust SE:
       Coef. Estimate   SE t-stat d.f. (Satt) p-val (Satt) Sig.
 (Intercept)    327.4 17.2  19.06        20.1      < 0.001  ***
      coachb    -42.3 32.1  -1.32         4.4      0.25268
      coachc    -57.9 18.7  -3.10        18.2      0.00615   **
  positionMF    122.9 14.4   8.54        39.1      < 0.001  ***
  positionST    143.5 21.3   6.74        32.3      < 0.001  ***
  positionWD    169.0 17.7   9.56        38.3      < 0.001  ***
    outcomeD     60.2 23.9   2.52        14.3      0.02408    *
    outcomeL     42.9 18.9   2.27        25.6      0.03175    *

--------------------------------------------------------
> compare_robust_results(lm_sprint, mixed_sprint, "Sprint")

 Model Comparison with Robust SE for: Sprint
============================================================

Linear Model with Robust SE:

t test of coefficients:

            Estimate Std. Error t value  Pr(>|t|)
(Intercept)  50.6798     5.3841  9.4129 < 2.2e-16 ***
coachb       -8.3362    13.2401 -0.6296   0.52942
coachc      -10.3049     7.3470 -1.4026   0.16176
```

```
positionMF   42.7157     6.9232  6.1699 2.185e-09 ***
positionST  107.2042    12.5367  8.5512 6.121e-16 ***
positionWD   75.9908     8.8386  8.5976 4.427e-16 ***
outcomeD      7.6536     8.7336  0.8763   0.38154
outcomeL     15.9343     7.5623  2.1071   0.03593 *
---
Signif. codes:  0 ‘***’ 0.001 ‘**’ 0.01 ‘*’ 0.05 ‘.’ 0.1 ‘ ’ 1
```

```
Mixed Effects Model with Robust SE:
        Coef. Estimate    SE t-stat d.f. (Satt) p-val (Satt) Sig.
 (Intercept)    50.66  6.09  8.319       21.06       <0.001  ***
      coachb    -8.27 15.51 -0.533        4.38       0.6196
      coachc   -10.12  7.69 -1.316       18.20       0.2044
  positionMF    42.24  6.41  6.588       39.15       <0.001  ***
  positionST   106.90 11.37  9.401       32.43       <0.001  ***
  positionWD    75.69  9.45  8.008       38.30       <0.001  ***
    outcomeD     7.87  8.01  0.982       14.33       0.3425
    outcomeL    15.95  9.24  1.726       25.60       0.0964    .
```
